# 2.5-Dimensional Structure Approach for Miniaturizing Flapping-Wing Air Vehicles

**DOI:** 10.3390/mi16111242

**Published:** 2025-10-31

**Authors:** Daisuke Ishihara, Motonobu Kimura, Ryotaro Suetsugu, Jyunpei Ueo, Naoto Ohira, Masakatsu Takagi, Kazuya Ishiba, Nagi Shirakawa, Ryusei Nishinohara, Masaaki Kimura

**Affiliations:** Department of Intelligent and Control Systems, Graduate School of Computer Science and Systems Engineering, Kyushu Institute of Technology, Iizuka, Fukuoka 8208502, Japan

**Keywords:** 2.5-dimensional (2.5-D) structure, microelectromechanical system (MEMS), piezoelectric, flapping-wing air vehicle (FWAV), biomimetics, insect flapping flight

## Abstract

In this study, we propose a 2.5-dimensional (2.5-D) structure approach for insect-mimetic flapping-wing air vehicles (FWAVs). The proposed approach includes design and fabrication methods. To our best knowledge, this study is the first one that develops a flapping system for FWAVs without any post-assembly of structural components. The proposed structure consists of a transmission, a supporting frame, and elastic wings. The transmission transforms the small translational displacement produced by a piezoelectric bimorph into a large rotational displacement of the wings. The size is reduced using the proposed design method. Then, the 2.5-D structure is fabricated using the proposed polymer MEMS micromachining method. The presented micro flapping system flaps the wing with a stroke angle and flapping frequency comparable to those of actual small insects using resonance. The results confirm that the proposed approach can miniaturize FWAVs.

## 1. Introduction

### 1.1. General Background

Insect flight is known for its stable hovering and agile maneuvering [1,2], which have evolved over time [3]. Mimicking these capabilities can lead to the realization of very small flapping-wing air vehicles (FWAVs). Insect-mimetic FWAVs have recently attracted much attention as miniaturized drones [4]. They have many potential applications, such as the exploration of areas that are difficult for conventional robots to penetrate (e.g., urban disaster areas). Flapping flight is a superior form of flight over fixed or rotary flight at low Reynolds number regimes in terms of maximizing the lift coefficient and minimizing the power coefficient [5].

### 1.2. Miniaturization of Air Vehicles

In 2005, the Defense Advanced Research Projects Agency (DARPA) started the Nano Air Vehicle Program, whose aim is to develop and demonstrate an extremely small (less than 7.5 cm) and ultralightweight (less than 10 g) air vehicle system with the potential to perform indoor and outdoor military missions [6]. Several studies on nano air vehicles have since been published [7]. Furthermore, pico air vehicles are defined as air vehicles with a maximum take-off mass of 500 mg and a maximum dimension of 5 cm [8]. They fall within the range of flying insects. Based on flying insects, this type of flyer can be reduced to about 1 cm or less in body length. However, it is difficult to further miniaturize flapping-wing pico air vehicles (FWPAVs). Our objective is to develop an approach for the further miniaturization of FWPAVs.

### 1.3. Flapping Mechanisms and Systems

There are two primary categories of driving mechanisms for FWAVs: motors and actuators. Motor-driven mechanisms rely on the integration of motors, reduction gears, and bars to create a functional flapping system [9]. They offer certain advantages, including cost-effectiveness, inconspicuousness, and reduced noise emissions. However, they are plagued by issues such as high energy consumption, suboptimal efficiency, and a lack of availability of miniature motors, making the miniaturization of motor-driven mechanisms a daunting task. Actuator-driven mechanisms are chosen to miniaturize the length and weight of FWAVs because of their lower complexity in integrating system components. These actuator-driven mechanisms use smart actuators such as piezoelectric [4,10,11,12], electromagnetic [13,14,15,16,17,18,19], dielectric elastomer [20,21], and electrostatic [22] actuators. The choice of actuator hinges on its capacity to deliver a high power density at the desired frequency. The detailed properties and characteristics of these actuators are summarized in ref. [23]. As shown, piezoelectric actuators are characterized by high stress generation and high energy density [24,25], as well as high response speed. Furthermore, they feature simplicity in structure and structural scalability [26,27]. Hence, in the context of pushing the limits of miniaturization in FWAV technology, which is the objective of this study, piezoelectric actuators are most promising. The piezoelectric actuator is combined with mechanical amplification called compliant mechanism [28] or transmission [29], since piezoceramic materials achieve relatively small strains.

The main components of insect-mimetic FWAVs are a power source, an actuator, a control unit, a transmission, wings, and a supporting frame [29]. According to the unsteady aerodynamic theory of insect flight, the wings’ flapping and pitching motions produce sufficient lift forces [30,31]. One priority in the construction of insect-mimetic FWAVs is to obtain a sufficient flapping motion or large wing displacement with kinematics similar to those of insects [13] since the fluid–structure interaction (FSI) can produce the characteristic pitching motion [8,32,33]. The transmission is responsible for the conversion of the actuator’s linear motion to a sufficient flapping motion. Here, we consider a flapping system that consists of a drivetrain (transmission and supporting frame) and an actuator.

### 1.4. Constraints Due to Fabrication Techniques

FWPAVs require novel fabrication methods [8]. Microelectromechanical system (MEMS) micromachining can be used to fabricate features with sizes on the order of micrometers. With increasing progress in MEMS technology, polymer micromachining has enabled the development of microstructures [34]. This experience might fill the gap between the material properties of traditional MEMSs and biological structures. However, their three-dimensional (3-D) capabilities are quite limited. It is extremely difficult to machine high-aspect-ratio components or fabricate hinged structures [8]. This issue has not been solved yet.

### 1.5. Previous Studies

The first prototype of an FWPAV that could create sufficient force to lift off was piezoelectric-driven [4]. The transmission was fabricated using mesoscopic manufacturing based on laser cutting, lamination, and folding. However, this approach may be difficult to apply for further miniaturization [13]. The previous FWPAVs fabricated using MEMS micromachining were electromagnetically driven [13,14,15]. In these prototypes, the structural components were fabricated using polymer MEMS micromachining. However, their post-assembly was necessary for constructing the flapping system. Furthermore, it had an added mass on its wings to obtain a large stroke angle [14]; however, such a mass might increase the difficulty of the FSI design of flexible wings [35]. On the contrary, in this study, a complex 2.5-dimensional (2.5-D) structure of an FWAV fabricable using MEMS micromachining without any post-assembly of structural components is designed, fabricated, and demonstrated to produce a large stroke angle without any added mass on the wings. Here, the term “2.5-D” represents the characteristic geometry of the proposed structure. The proposed structure consists of laminated thin layers only, where each layer is freely shaped in the in-plane directions, while the shape is uniform in the out-of-plane direction and the number of layers is limited due to the fabricability. In this study, because of these constraints imposed on the geometry, we call the dimension “2.5-D” as the intermediate of “2-D” and “3-D”.

### 1.6. Objective in This Study

In this study, we propose a 2.5-D structure approach for FWAVs. The proposed approach includes the design and fabrication methods of a 2.5-D structure of insect-mimetic FWAVs. A size reduction in this structure is achieved using the proposed design method. Complex structures are fabricated using the proposed polymer micromachining method without any post-assembly. Innovative claims include that (a) to our best knowledge, there are no studies on prototyping FWAVs that are fabricable using MEMS micromachining except our study and refs. [13,14,15]; (b) furthermore, only our study does not require any post-assemply process to develop the drivetrain, and (c) a piezoelectric-driven flapping system that uses resonance is developed and a wing without any added mass is flapped with a large stroke angle and flapping frequency comparable to those of actual small insects. The results confirm that the proposed 2.5-D structure approach can be used to miniaturize FWPAVs.

## 2. Two-Point-Five-Dimensional Structure Approach for Insect-Mimetic FWAVs

### 2.1. Concept and Basic Design

#### 2.1.1. Concept of 2.5-D Structure Approach

**Figure 1:**
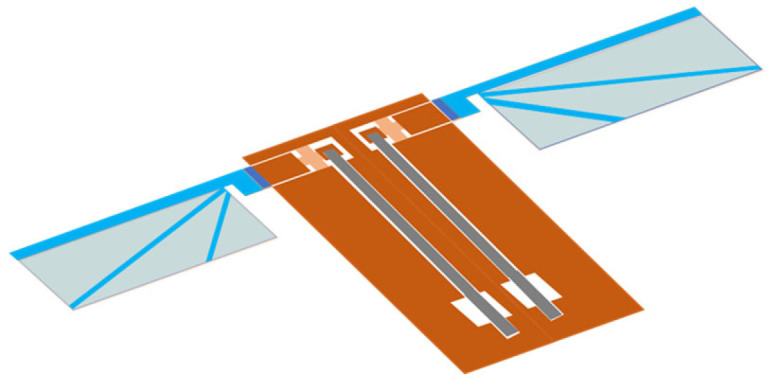
Insect-mimetic FWAV with complete 2.5-D structure or MEMS flyer. The brown part is the drivetrain, which consists of a transmission, a supporting frame, a wing attachment and an actuator connection. The blue part is the micro wing, and the gray part is the piezoelectric bimorph actuator. The flapping system consists of the drivetrain and the actuators.

Figure 1 shows a conceptual view of an insect-mimetic FWAV based on the proposed 2.5-D structure approach or MEMS flyer. The FWAV has a complete 2.5-D structure and can thus be fabricated using MEMS micromachining based on photolithography. The proposed approach transforms a 3-D insect structure into a 2.5-D structure. For this transformation, the structure is designed such that it replicates the desired function using the same mechanism. The design problem is a nonlinear coupled problem that includes multiphysics and micromachining, and it is solved using computational methods, leading to computational biomimetics.

#### 2.1.2. Transmission Using Geometrical Nonlinearity

**Figure 2:**
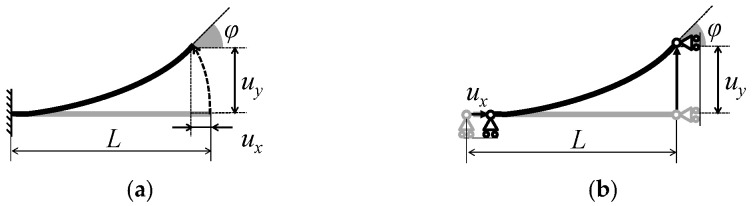
Transmission mechanism based on geometrical nonlinearity of large bending of cantilever beams. (**a**) Beam model for T-shaped hinge, where left end is fixed to supporting beam. (**b**) Beam model for I-shaped hinge, where left end is connected to actuator. A bold line represents the beam corresponding to the elastic hinge. *L* is the length of the beam, *u_x_* is the displacement produced by the actuator, *u_y_* is the deflection of the hinge, and *φ* is the deflection angle.

**Figure 3:**
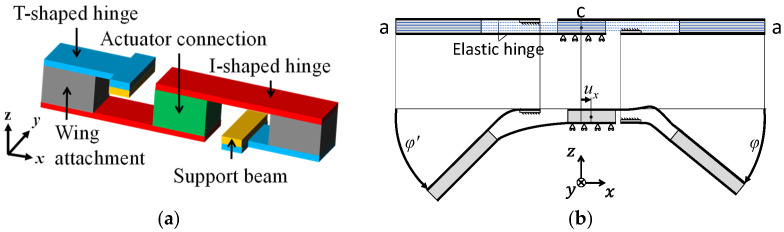
Basic design of transmission. (**a**) Three-dimensional view. (**b**) Sectional view.

Figure 2 shows the proposed transmission mechanism. The small translational displacement ux from the actuator to elastic hinges is transformed into a large rotational displacement φ for large flapping motions using the geometrical nonlinearity of the large bending of cantilevers. Figure 2a,b show the beam models for the T- and I-shaped hinges in Figure 3a, respectively.

Figure 3a shows a basic design of this transmission. The transmission consists of a pair of elastic hinges (T- and I-shaped elastic hinges), a support beam, a wing attachment, and an actuator connection. In this study, a piezoelectric bimorph is adopted as the actuator since it is suitable for MEMS fabrication. Figure 3b shows a sectional view of this transmission. The piezoelectric bimorph imposes a small translational displacement at point C and produces a large bending of the elastic hinges, which leads to sufficient flapping motions.

In a two-wing transmission, the rotational moments from the left- and right-hand sides of the elastic hinges act on the actuator connection. These moments are in opposite directions and thus cancel out. In contrast, in a one-wing transmission, the rotational moment from the left- or right-hand side of the elastic hinges acts on the actuator connection, and the piezoelectric bimorph supports the actuator connection. Hence, the piezoelectric bimorph must have sufficient torsional stiffness.

### 2.2. Fabrication Process of Polymer MEMS Micromachining

**Figure 4:**
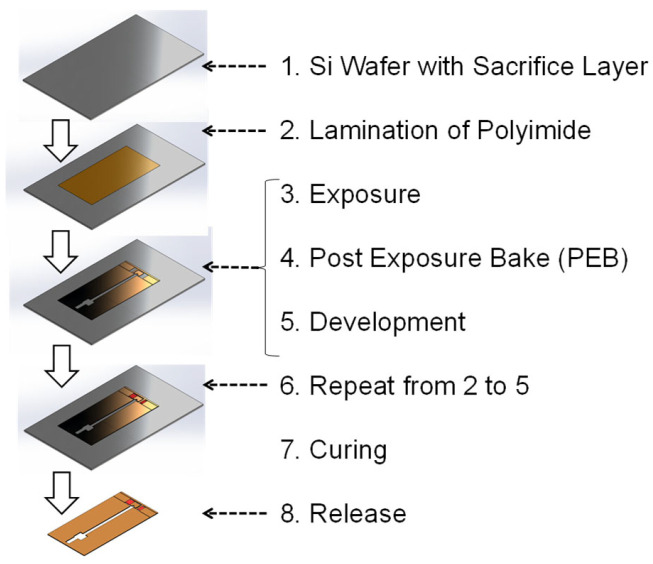
Fabrication process of polymer micromachining.

Figure 4 shows the microfabrication steps of the 2.5-D structure using a liquid solution and adhesive sheets of photosensitive polyimide (PI) precursor. The PI precursor is laminated, exposed, and developed on the substrate (a Si wafer with a Cu sacrificial layer created using Cu vapor deposition). This process is repeated until the specified 2.5-D structure is constructed. Then, the 2.5-D structure is cured to imidize the precursor and convert it to PI. All layers are laminated without curing to tightly couple them. Finally, the 2.5-D structure is released from the substrate by etching the Cu sacrificial layer using a ferric chloride aqueous solution.

The first PI layer, whose thickness is a few micrometers, is formed on the Cu sacrificial layer via the spin-coating of a photosensitive PI precursor liquid solution. This layer serves as a coupling agent between the Cu sacrificial layer and the photosensitive PI precursor adhesive sheets and forms wing membranes. On the PI membrane, the photosensitive PI precursor adhesive sheets (thickness per sheet: a few dozen micrometers) are laminated to form the beam, plate, and shell structures.

**Figure 5:**
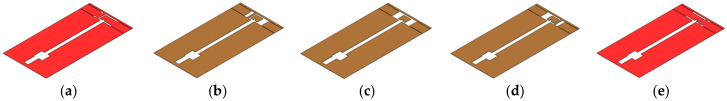
In-plane shape of each layer for two-wing drivetrain. The layers shown in panels (**a**–**e**) are laminated in this order and, respectively, form the lower T- and I-shaped elastic hinges, beam for supporting the lower T-shaped hinge, gaps between the lower and upper hinges, beam for supporting the upper T-shaped hinge, and upper T- and I-shaped elastic hinges.

**Figure 6:**
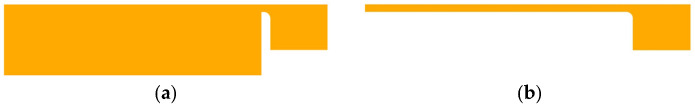
In-plane shape of each layer for wing. (**a**) Layer that includes wing membrane. (**b**) Layer that includes leading edge beam.

Figure 5 and Figure 6 show the plane structures of the layers for the drivetrain and wing, respectively. In the proposed FWAV, the first layer is made as shown in Figure 5a and Figure 6a using the PI membrane (thickness: a few micrometers), and the following layers are made as shown in Figure 5b–e for the drivetrain and Figure 6b for the wing using the PI sheets (thickness per sheet: a few dozen micrometers).

### 2.3. Shape Design for Fabricability

Various stresses in polymer MEMS micromachining processes, such as lamination, development, cooling after post-exposure baking, and curing, cause cracks in the brittle PI precursor. Hence, this section presents the shape design used to reduce these stresses.

#### 2.3.1. Rounding of Concave Corners

**Figure 7:**
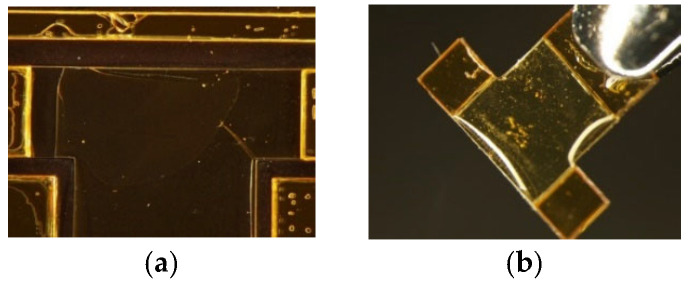
Concave corners (**a**) without rounding and (**b**) with rounding.

Figure 7a shows a crack that formed in the development process for prototyping a pair of T-shaped elastic hinges. The crack formed at the sharp concave corner because of the stress concentration there. Hence, the corner was rounded to reduce stress concentration. With this change, the 2.5-D structure was successfully fabricated, as shown in Figure 7b.

#### 2.3.2. Rounding of and Reduction in Number of Convex Corners

**Figure 8:**
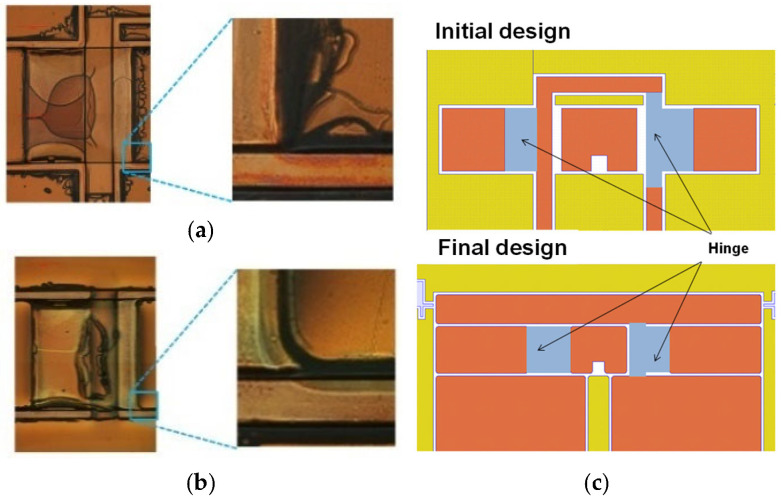
(**a**) Failure of adhesion at sharp convex corner. (**b**) Rounding of convex corner (improves adhesion at corner). (**c**) Reduction in number of convex corners.

As shown in Figure 8a, incomplete adhesions frequently occur at sharp convex corners, producing protrusions at the corners and leading to cracks due to the stress concentration there. Hence, the convex corners were rounded, as shown in Figure 8b. Furthermore, the number of convex corners was reduced, as shown in Figure 8c.

#### 2.3.3. Support Domain for Each Layer in Lamination Process

In the white domain in Figure 8c, when each layer is laminated, out-of-plane deformation occurs because of the cavity under it, leading to failure. Hence, the yellow domain was used to reduce the white domain and support the layer. Note that this domain is discarded in the release process.

## 3. Structural Design for Size Reduction

### 3.1. Design Problem and Method

#### 3.1.1. Design Problem

**Figure 9:**
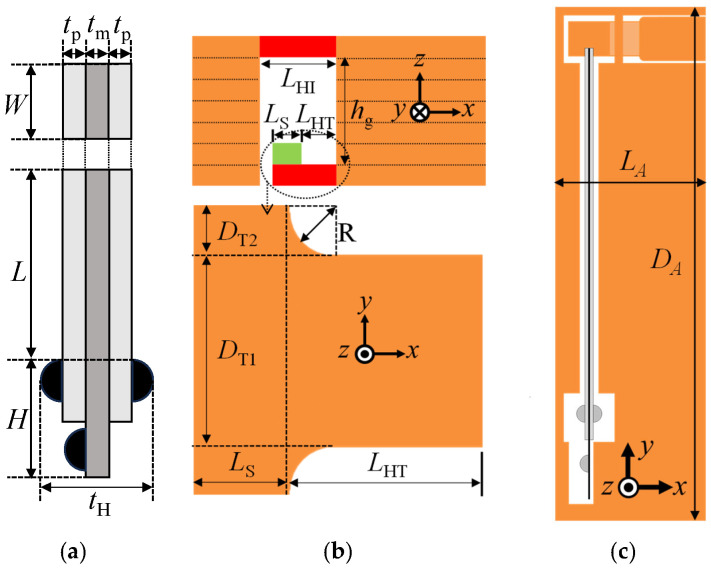
Primary design parameters of flapping system. (**a**) Piezoelectric bimorph, (**b**) transmission and hinges, and (**c**) supporting frame.

The structural design problem considered here is the reduction in the size of the flapping system while maintaining a sufficient stroke angle. Figure 9 shows the dimensions of the flapping system, which were used as the major design parameters. Table 1 shows the values of these parameters for large and small flapping systems. The values for the large flapping system were used for the initial design solution. The thickness of each layer was limited to 26 or 40 μm (photosensitive PI precursor adhesive sheets with these thicknesses were used in this study).

#### 3.1.2. Structural Design Method

A structural design method is proposed to solve the above design problem. The proposed method consists of a basic design based on 2-D structural models and a detailed design based on 3-D solid models. In the basic design, the representative length or length of the piezoelectric bimorph *L*, shown in Figure 9a, is first reduced, and then the dimensions of the transmission and hinges, shown in Figure 9b, are reduced in accordance with *L*. In the detailed design, the dimensions of the supporting frame, shown in Figure 9c, are reduced in accordance with the basic design.

In the basic design, each design solution candidate is evaluated using FE analyses for the 2-D structural models to reduce the computational cost for numerous search points in the design parameter space. First, we use the beam model and nonlinear FE analysis to evaluate the stroke angle while L and the hinge dimensions are reduced. The beam model describes the coupling of the drivetrain and the piezoelectric bimorph (see next section for details). The nonlinear FE analysis considers the geometrical nonlinearity of the beam model since the elastic hinges exhibit large bending. Second, we use the plane stress model for the T-shaped elastic hinge and linear FE analysis to evaluate the plane stress due to heat shrinking in the curing process while the hinge dimensions are reduced.

In the detailed design, each design solution candidate is evaluated using FE analyses for the 3-D solid models based on their basic design solutions to accurately predict their 3-D elastic behaviors. First, we use the 3-D solid model for the drivetrain and nonlinear FE analysis. In the 2-D beam model, the supporting frame is ideally described as a fixed boundary. In contrast, in the 3-D solid model, the supporting frame and the other parts of the drivetrain are described as a 3-D elastic body to evaluate the strength and stroke angle while the dimensions of the supporting frame are reduced. Nonlinear FE analysis is used for this model because of the large deformation of the transmission. Second, we use the 3-D solid model for the flapping system and FE mode analysis to predict the resonance in the piezoelectric-driven insect-mimetic FWAV. In this model, the drivetrain and the piezoelectric bimorph are described as a single elastic continuum because of their tight coupling.

#### 3.1.3. Two-Dimensional Beam Model for Coupling of Transmission and Piezoelectric Bimorph

**Figure 10:**
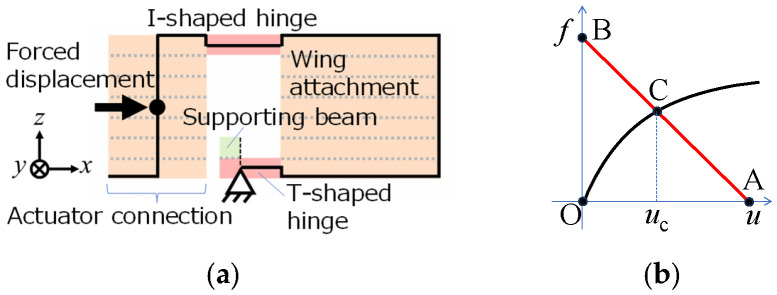
(**a**) Two-dimensional beam model for coupled transmission and piezoelectric bimorph. (**b**) Relationship between forced displacement and reaction force at actuator connection of transmission (black curve) and relationship between displacement and generated force at tip of piezoelectric bimorph (red line).

Figure 10a shows the proposed beam model that describes the bending behavior of the transmission. The transmission is expressed using beam elements. The beam elements for the elastic hinges are located at their neutral plane. Their material properties are set to those of PI (Young’s modulus: 2.5 GPa; Poisson’s ratio: 0.289). The material properties of the other parts (wing attachments and actuator connection) are set such that they sufficiently behave as a rigid body. The support beams are modeled as a fixed support.

The transmission and the piezoelectric bimorph satisfy the equilibrium of forces between them. Equilibrium point C in Figure 10b can be estimated as follows: Let the generating force of the piezoelectric bimorph in the *x* direction at the actuator connection be denoted as *f*_p_ and the elastic reaction force of the transmission in the *x* direction at the actuator connection be denoted as *f*_t_. Then, the x displacement *u* at the actuator connection is determined as the solution *u*_C_ of the equilibrium equation *f*_t_ = *f*_p_.

The relationship between *u* and *f*_t_ is given using the nonlinear FE analysis for the 2-D beam structure model shown in Figure 10a. The relationship between *u* and *f*_p_ is given as follows. Point A in Figure 10b is the bending displacement *δ* of the piezoelectric bimorph without any transverse force. Its analytical solution can be given as [36]

(1) $$\delta =\frac{3L^{2}}{2(t_{m}+2t_{p})}\frac{2\alpha \beta d_{31}{\left(1+\beta \right)}^{2}}{\alpha^{2}B^{4}+2\alpha (2\beta +3\beta^{2}+2\beta^{3})+1}\frac{V}{t_{p}},$$

where

(2) $$\alpha =E_{\mathrm{pm}}/E_{p},\;\beta =(t_{m}+t_{p})/t_{p},$$

and *t*_m_ is the thickness of the shim plate, *t*_p_ is the thickness of each PZT layer, *L* is the length of the bimorph, *V* is the applied voltage, *E*_p_ is the Young’s modulus of the PZT material (61 GPa), *E*_m_ is the Young’s modulus of the shim material (140 GPa), *E*_pm_ is the Young’s modulus of the composite material of each PZT layer and shim plate, and *d*_31_ is the piezoelectric strain constant (1.98 × 10^−10^ m/V). Point B in Figure 10b is the transverse force *F* acting on the tip to give *δ*. Its analytical solution can be expressed, under a Bernoulli–Euler beam assumption, as

(3) $$F=(3E_{\mathrm{pmp}}I/L^{3})\delta$$

where *E*_pmp_ is the Young’s modulus of the composite material of both PZT layers and the shim plate and *I* is the second moment of the area. The force *F* given by Equation (3) is called the blocking force. The response of the piezoelectric bimorph, or the relationship between *u* and *f*_p_, can be given as the straight line between points A and B, as shown in Figure 10b. The sensitivity of the response of the piezoelectric bimorph can be expressed as the bending stiffness of the bimorph 3*E*_pmp_*I*/*L*^3^ from Equation (3).

### 3.2. Basic Design Results

This section presents the basic design results obtained using the structural design method described in Section 3.1. The large flapping system in Table 1 is used as the initial design solution. For simplicity, one-wing flapping systems are considered here. The beam model and nonlinear FE analysis in Section 3.1 are used in Section 3.2.1, Section 3.2.2 and Section 3.2.3, and the plane stress model and linear FE analysis are used in Section 3.2.4.

#### 3.2.1. Representative Length of Flapping System

First, the representative length of the flapping system or the length of the piezoelectric bimorph *L* is reduced. The width of the bimorph *W*, the thickness of the bimorph 2*t*_p_+ *t*_m_, and the thickness of the PI sheet are set to 2 mm, 0.5 mm, and 26 μm, respectively, which are the minimum values available at this time. The gap between the T- and I-shaped hinges *h*_g_ is reduced to 130 μm since the number of PI sheets for the gap is initially five, following the large flapping system. The applied voltage *V* is fixed at 200 V throughout the following design process.

**Figure 11:**
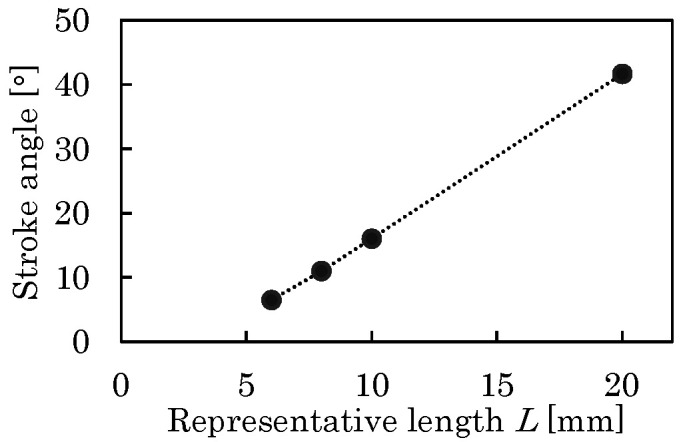
Relationship between actuator length *L* and stroke angle *Φ*.

Figure 11 shows the relationship between *L* and the stroke angle *Φ*. As shown, *Φ* changes linearly in the range of *L* considered here. It is reduced from 42° to 16° as *L* is reduced from 20 to 10 mm. Under the assumption that the resonant drive can compensate *Φ* for this level of reduction, the representative length of the small flapping system is set to *L* = 10 mm.

#### 3.2.2. Elastic Hinge Lengths

**Figure 12:**
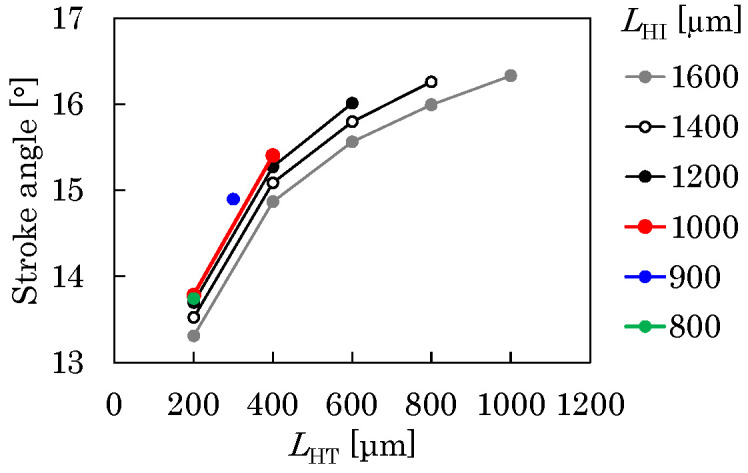
Relationship among hinge lengths *L*_HT_ and *L*_HI_ and stroke angle *Φ*.

Following a 50% reduction in *L*, the lengths of the T- and I-shaped elastic hinges, denoted as *L*_HT_ and *L*_HI_, respectively, are reduced. The geometrical interference constraint *L*_HI_ − *L*_HT_ − *u*_C_ >= *L*_s_ is imposed (see Figure 9b). Figure 12 was obtained under this condition, where *u*_C_ was assumed to be 100 μm. As shown, in the case of reducing (*L*_HI_, *L*_HT_) from (1.8 mm, 1.0 mm) to (1.0 mm, 0.4 mm), which is almost the same reduction ratio as that for *L*, the stroke angle is 15.4°, which is almost the same value as that before the reduction. Hence, *L*_HI_ and *L*_HT_ are set to 1.0 and 0.4 mm, respectively.

#### 3.2.3. Gap Between T- and I-Shaped Hinges

The gap *h*_g_ between the T- and I-shaped hinges should be sufficiently large to avoid sticking due to the surface tension of the developing liquid. On the other hand, the probability of fabrication failures increases with an increasing number of layers. Furthermore, *h*_g_ influences *Φ*. Hence, the relationship between *h*_g_ and *Φ* is investigated to facilitate the selection of *h*_g_.

**Figure 13:**
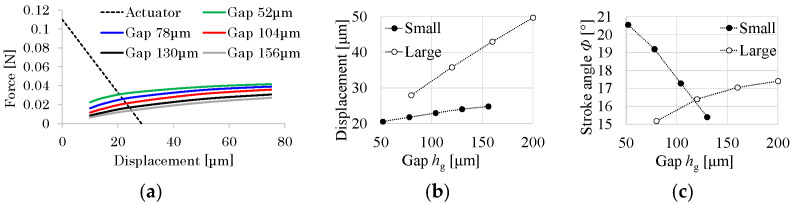
Relationships between gap *h*_g_ and drivetrain performance. (**a**) Displacement and force for small drivetrain, where solid lines correspond to transmission and dotted line corresponds to actuator. (**b**) Gap and displacement at equilibrium point. (**c**) Gap and stroke angle at equilibrium point.

*h*_g_ is set to 26 μm (thickness of each layer) × *n* (*n* = 2, 3, …). All other dimensions are set as described in the previous section. Figure 13a shows the relationships among the displacements, generating the force of the piezoelectric bimorph and reaction force of the transmission at the actuator connection in the *x* direction. As shown, the displacement of any reaction force increases monotonically as *h*_g_ increases because *h*_g_ corresponds to the arm length of the moment for the bending of the hinges.

Figure 13b shows the relationship between *h*_g_ and *u*_C_ for the small drivetrain, derived from Figure 13a, and large drivetrain (see Table 1). The slope for the small drivetrain is much smaller than that for the large drivetrain since the bending stiffness in Equation (3) for the small drivetrain is 11 times larger than that for the large drivetrain. Hence, as shown in Figure 13c, as *h*_g_ increases, *Φ* decreases for the small drivetrain but increases for the large drivetrain due to the geometrical relationship between *u*_C_/*h*_g_ and *Φ*. For *h*_g_ = 130 μm (*n* = 5), which corresponds to the above section, the stroke angle is 15.4°, and for *h*_g_ = 104 μm (*n* = 4), the stroke angle is 17.3°. Furthermore, the ratio of *h*_g_ to the hinge length for *h*_g_ = 104 μm (*n* = 4) is close to that for the large drivetrain. Hence, *h*_g_ is set to 104 μm (*n* = 4).

#### 3.2.4. Stress Reduction in Curing Process

In the curing process, failure of the supporting beam of the T-shaped elastic hinge can occur due to material shrinking. Hence, the sizing optimization of the T-shaped elastic hinge is conducted.

**Figure 14:**
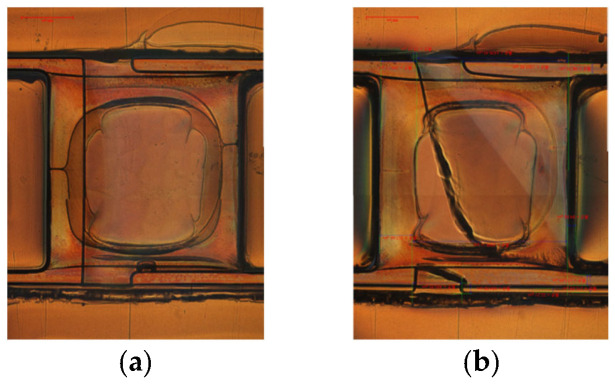
Failure of T-shaped hinge corner for *D*_T2_ = 100 μm, *R* = 50 μm, and thickness of 40 μm (**a**) before and (**b**) after curing.

First, the large flapping system is considered as the reference case. An image of this type of failure is shown in Figure 14. A comparison between the in-plane images of the T-shaped elastic hinge before and after curing indicates that tension in the downward direction is imposed on the lower end of the supporting beam due to the in-plane shrinking of the supporting frame, causing the in-plane deformation of the T-shaped elastic hinge. As a result, a crack forms at the corner due to stress concentration.

**Figure 15:**
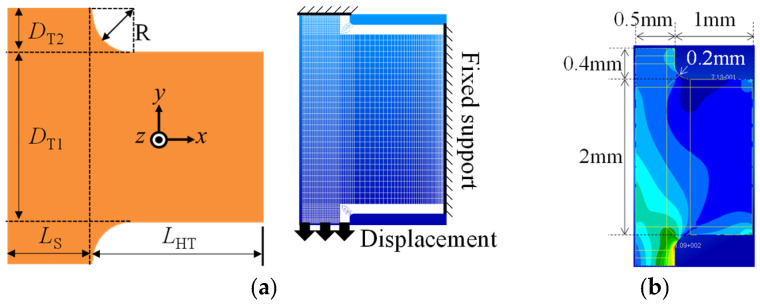

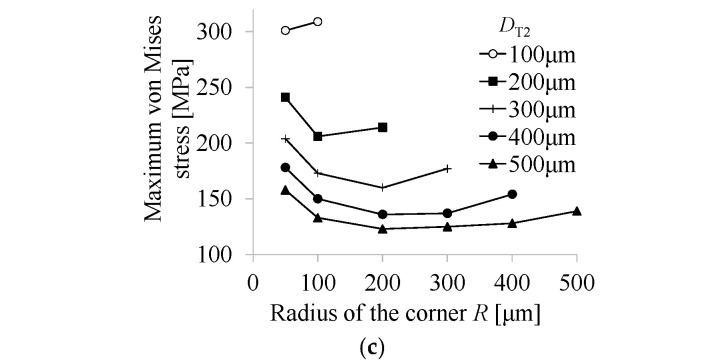
Stress due to shrinking of supporting frame in curing process for large drivetrain (Young’s modulus: 3 GPa; Poisson’s ratio: 0.3). (**a**) Plane stress modeling of T-shaped elastic hinge. (**b**) Von Mises stress distribution for large drivetrain. (**c**) Maximum von Mises stress for *R*, where *D*_T2_ is set to 100, 200, 300, 400, and 500 μm.

To simulate this phenomenon, it is formulated as the in-plane stress problem shown in Figure 15a. The forced displacement imposed on the lower end of the support beam is set to −32 μm in the *y* direction based on Figure 15a. For PI, Young’s modulus is set to 3 GPa and Poisson’s ratio is set to 0.3. The thickness of the plate is set to 40 μm. *D*_T2_ and *R* are set to 100 and 50 μm, respectively, and the other dimensions are set as shown in Figure 15b. The linear FE analysis results show that the maximum von Mises stress *σ*_m_ is at the lower corner, similar to the actual failure. This result validates the present analysis method.

The failure criterion is as follows. In the actual fabrication, no failure occurs for *D*_T2_ = 500 μm and *R* = 50 μm. In the FE analysis for this case, *σ*_m_ = 158 GPa at the corner. Hence, this value is used as the failure criterion. Figure 15c shows *σ*_m_ for various *D*_T2_ and *R* values. *D*_T2_ = 400 and 500 μm give admissible solutions. Here, *D*_T2_ = 400 μm is selected since a smaller *D*_T2_ leads to the formation of a smaller in-plane gap between the hinge and the supporting frame, which reduces the probability of failure (see Section 2.3.3). Furthermore, *R* = 200 μm is selected since it minimizes *σ*_m_. In summary, the dimensions of the elastic hinge for the large drivetrain are *L*_s_ = 500 μm, *L*_HT_ = 1.0 mm, *D*_T1_ = 2.0 mm, *D*_T2_ = 400 μm, and *R* = 200 μm, as shown in Figure 15b and Table 1. This figure also shows the von Mises stress distribution, where *σ*_m_ is at the corner.

**Figure 16:**
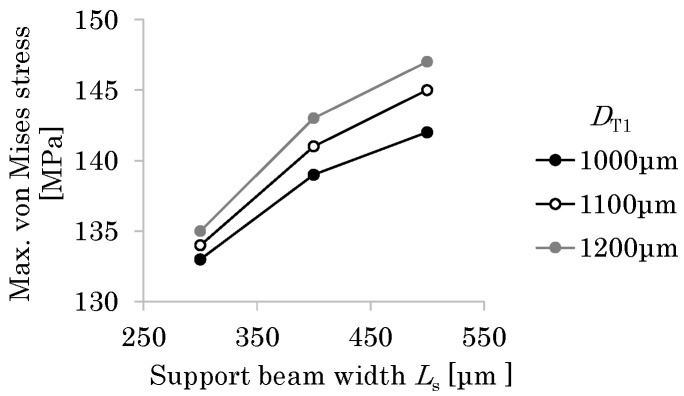
Relationship among design parameters and stress for small drivetrain.

Next, the sizing optimization of the small drivetrain is conducted based on the large drivetrain. For the large drivetrain, the maximum von Mises stress (*σ*_m_ = 136 MPa) is at the corner of the T-shaped hinge. Here, this value is set as the failure criterion. *D*_T2_ and *R* are fixed and *L*_HT_ is set to 0.4 mm following the previous section, while *L*_s_ and *D*_T1_ are considered as the design parameters. For PI, Young’s modulus is set to 3 GPa and Poisson’s ratio is set to 0.3. The thickness of the plate is set to 26 μm. The tension imposed on the lower end of the support beam is set to 20 μm, derived by multiplying the tension for the large flapping system (32 μm) by the ratio of the supporting frame length of the small drivetrain to that of the large drivetrain. Figure 16 shows the relationship among *L*_s_, *D*_T1_, and *σ*_m_. *L*_s_ = 300 μm satisfies the failure criterion for any *D*_T1_. Hence, *L*_s_ = 300 μm is selected. Furthermore, *D*_T1_ = 1000 μm is selected since it minimizes *σ*_m_.

### 3.3. Detailed Design Results

#### 3.3.1. Supporting Frame

**Figure 17:**
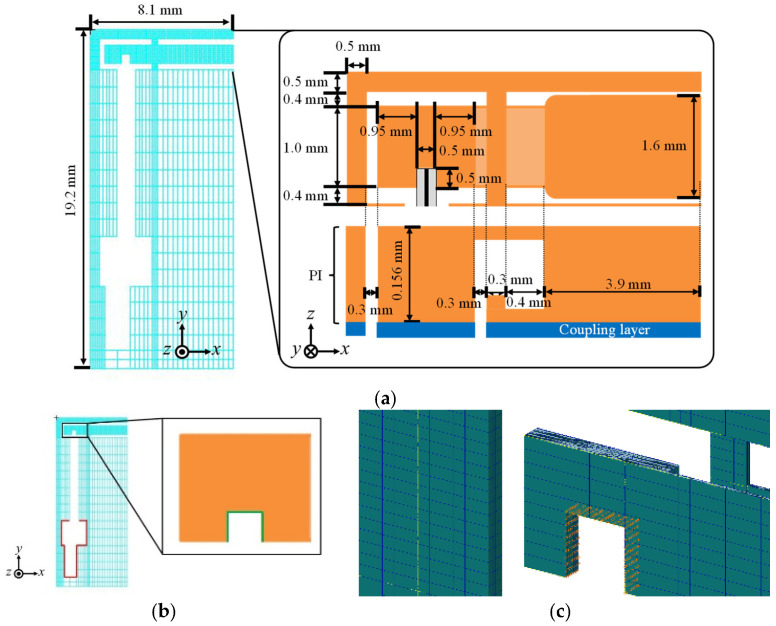
Three-dimensional model for detailed design. (**a**) Initial design solution. (**b**) Boundary conditions (red lines: fixed support; green lines: forced displacement). (**c**) Close-up view of mesh around boundary, where forced displacement is imposed on nodes colored orange.

In the detailed design, the width *L*_A_ is considered as the design parameter since it determines the overall size and strength of the flapping system. Figure 17a shows the initial design solution, where the size of the large drivetrain is reduced following the above basic design results.

The drivetrain is made of PI (Young’s modulus: 2.5 GPa; Poisson’s ratio: 0.289). The coupling layer is considered in the modeling for accuracy. For this layer, Young’s modulus is set to 3 GPa, Poisson’s ratio is set to 0.3, and the thickness is set to 7.3 μm (based on observations).

Figure 17b,c show the boundary conditions. The fixed support and the forced displacement are imposed on the surfaces of the supporting frame that adhere to both ends of the piezoelectric bimorph. The forced displacement is determined using the beam model described in Section 3.1.3 as −25 μm in the *x* direction. Hexahedral quadratic elements are used for the mesh. We consider the following three design candidates.

Case 1: From the initial design solution, the width of the actuator attachment (2.4 mm) is reduced by half to 1.2 mm following the reduction rate of the characteristic length *L*, while the gaps among the supporting frame, transmission, and piezoelectric bimorph are maintained, leading to the design solution candidate *L*_A_ = 7.5 mm.

**Figure 18:**
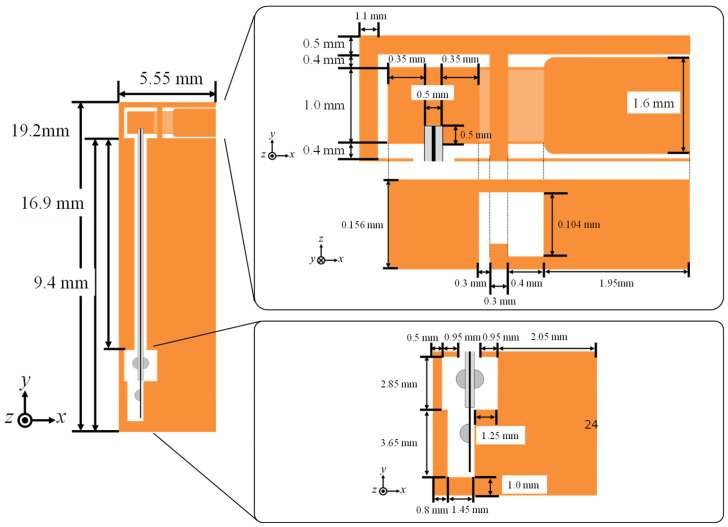
Detailed model of final design for small drivetrain.

Case 2: From Case 1, the width of the wing attachment (3.9 mm) is reduced by half to 1.95 mm, leading to the design solution candidate *L*_A_ = 5.55 mm, as shown in Figure 18.

Case 3: From Case 2, the width of the wing attachment is further reduced such that *L*_A_ is half the size of that for the large drivetrain, leading to the design solution candidate *L*_A_ = 4.45 mm.

**Figure 19:**
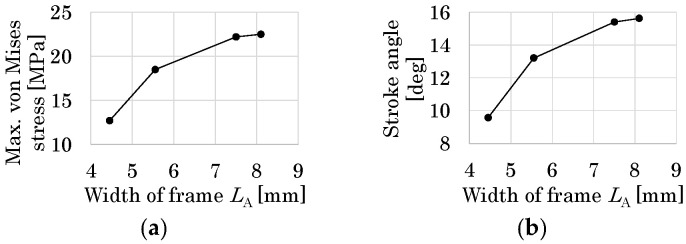
(**a**) Relationship between width of frame *L*_A_ and maximum von Mises stress. (**b**) Relationship between *L*_A_ and stroke angle.

For all cases, the maximum von Mises stress is at the corner of the T-shaped elastic hinges and is sufficiently smaller than the yield stress of PI (109 MPa), as shown in Figure 19a. As shown in Figure 19b, the reduction in stroke angles is significant from Case 2 to Case 3. Hence, the design solution candidate *L*_A_ = 5.55 mm (Case 2) is selected as the final design solution.

#### 3.3.2. Vibration Modes and Natural Frequencies

**Figure 20:**
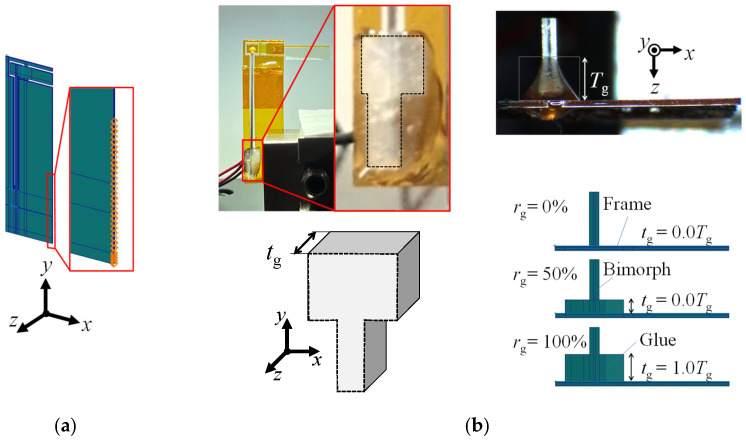
(**a**) Three-dimensional solid modeling of coupled drivetrain and bimorph. (**b**) Solid modeling of glue part based on observations.

Insects flap their wings at the fundamental frequency of their thorax to reduce the energy requirements of flight [37]. Mimicking this mechanism, in this study, the flapping system, as a monolithic vibration system that consists of the drivetrain and the piezoelectric bimorph, is resonantly driven. Here, FE mode analysis is conducted for the large flapping system to clarify the proposed resonant drive. Figure 20a shows the 3-D solid modeling of the large flapping system using FE discretization with hexahedral quadratic elements. The fixed support is imposed on the same edge as the corresponding experiment. The bimorph is glued to the supporting frame at both ends. The weight of this part affects the dynamics of the whole system, but the shape of this part does not affect the dynamics since this part behaves as a rigid body. Hence, the glue part is modeled as a rectangular solid, as shown in Figure 20b, where the glue occupancy *r*_g_ = *t*_g_/*T*_g_ (*t*_g_ is the thickness of the rectangular solid and *T*_g_ is the maximum height of the glue part) is used as the geometrical parameter. Table 2 summarizes the material properties. The in-plane area of this part (dotted line in Figure 20b) is set based on the actual space for setting the bimorph’s end.

**Figure 21:**
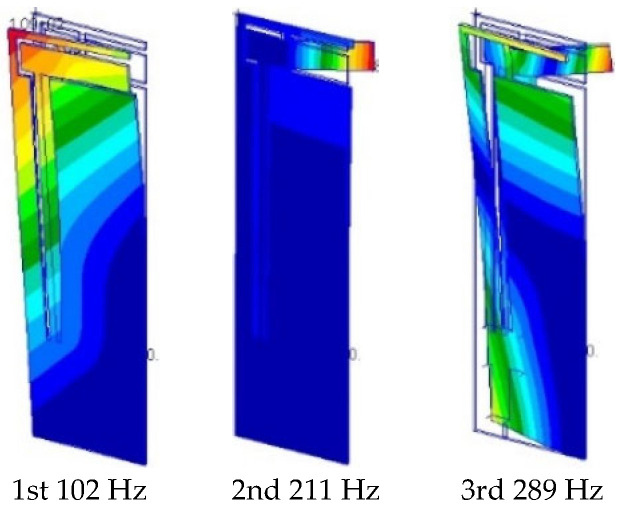
Vibration modes and natural frequencies.

Figure 21 shows the results from the FE mode analysis using *r*_g_ = 50% and the maximum values of the material properties for the epoxy resin in Table 2. Note that the *n*-th-mode shapes (*n* = 1, 2, or 3) for *r*_g_ values ranging from 0% to 100% and the different material properties for the epoxy resin in Table 2 are similar to each other. As shown, the first mode corresponds to the out-of-plane bending of the whole flapping system in the longitudinal direction and the second mode corresponds to the out-of-plane bending of the elastic hinges in the span direction. These vibration modes can produce a base excitation of the wing attachment such that it flaps the outer wing.

**Figure 22:**
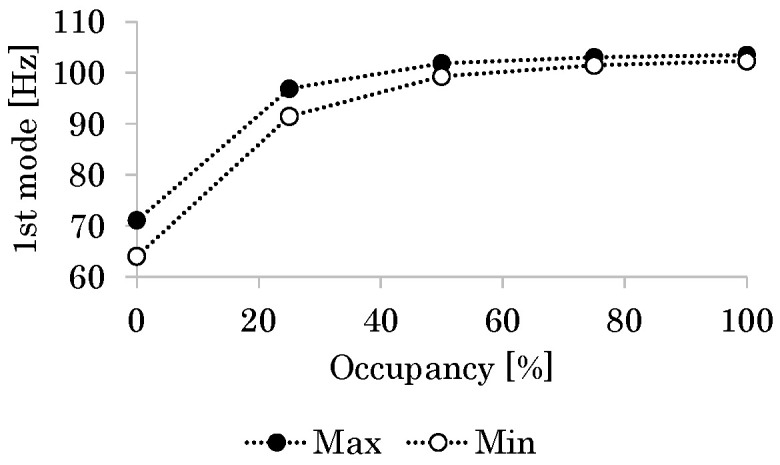
Relationship between glue occupancy *r*_g_ and first-mode natural frequency *f*_n_. “Max” and “Min” denote the maximum and minimum values of the material properties, respectively.

Figure 22 shows the relationship between *r*_g_ and the first-mode natural frequency *f*_n_. As *r*_g_ increases or the coupling between the bimorph and the frame strengthens, *f*_n_ increases because of the increasing stiffness of the flapping system around the fixed support. The available frequencies for the resonant drive are very close to the flapping frequencies of actual small insects (those with indirect flight muscles). These results indicate that the flapping system based on the resonant drive is a feasible solution.

## 4. Micromachining and Testing of Flapping Systems

### 4.1. Occurrence of Cracks and Failures in Fabrication and Their Prevention

Photosensitive PI precursor materials are brittle before curing. Hence, design and fabrication methods that avoid the failures caused by stresses during the fabrication process are necessary, especially for multilayer structures that include multiple cavities (e.g., the proposed 2.5-D structure). In this section, fabrication methods are proposed to avoid failure problems in the fabrication process of the proposed 2.5-D structure.

#### 4.1.1. Bubble Control in Development Process

**Figure 23:**
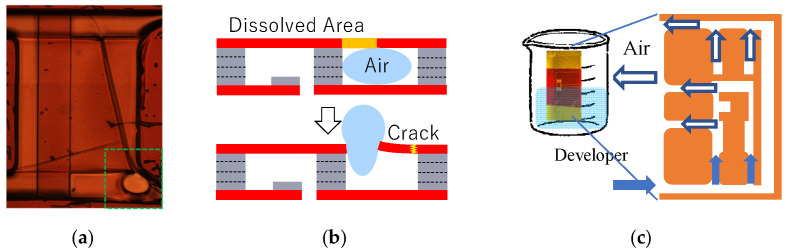
(**a**) Crack due to air bubbles passing through side of elastic hinges in development process (see the area surrounded by green dotted lines). (**b**) Schematic diagrams of crack formation mechanism. (**c**) Bubble control method, where capillary action of developer through flow channel is used to exhaust air.

In the development process, air bubbles can cause cracks in elastic hinges, as shown in Figure 23a. Consider a cavity between the two layers that form the elastic hinges. As schematically shown in Figure 23b, if air is encapsulated in the cavity, then it will pass through the side of the elastic hinge as a bubble in the development process. The resulting pressure causes a crack in the elastic hinge.

The bubble control method is used to prevent this failure, as shown in Figure 23c. The inlet of the flow channel in the chip is immersed in the developer. Then, capillary action introduces the developer into the flow channel to suffuse the flow channel with the developer. Finally, the air in the channel is exhausted from the outlet of the flow channel in the chip before development is completed. In this method, the flow channel is designed such that the cavity for the elastic hinge is located at any point of the flow channel between the inlet and outlet.

#### 4.1.2. Release Progress Control

**Figure 24:**
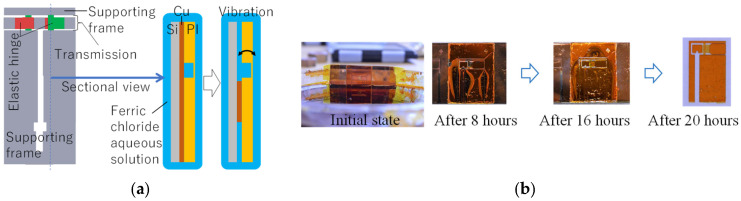
Release progress control. (**a**) Issue in release process using beaker method. (**b**) Release process under release progress control.

The beaker method with a ferric chloride aqueous solution is used to release the structure from the Cu sacrificial layer. In this process, if the lower supporting frame remains while the other part is released, then the flow of the solution liquid causes vibration of the released part around the elastic hinge, as schematically shown in Figure 24a, leading to the failure of the elastic hinge. Release progress control is used to prevent this failure. Initially, the end of the lower supporting frame is immersed in the solution liquid to release only the lower supporting frame, as shown in Figure 24b. After this release has sufficiently progressed, the whole chip is immersed in the solution liquid such that all the parts are released at almost the same time. As shown, the release progress is controlled and the specimen is successfully released.

#### 4.1.3. Cooling Process After Post-Exposure Baking

**Figure 25:**
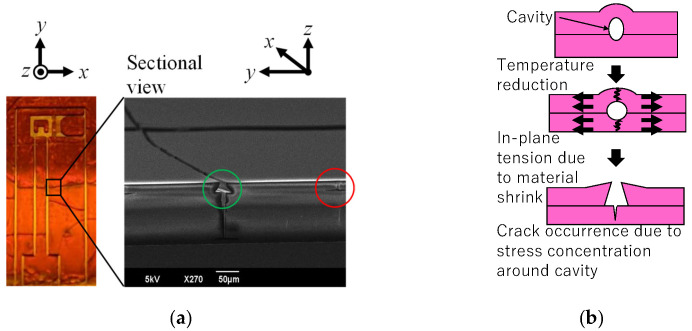
(**a**) Crack formation due to heat stress during temperature decrease process after post-exposure baking (green circle: fully developed crack; red circle: less-developed crack) and (**b**) crack mechanism.

As shown in Figure 25a, cracks frequently form in the cooling process after post-exposure baking. The red circle in this figure indicates a small crack that formed around the small cavity between two layers. It seems that the larger crack, indicated by the green circle, developed from this type of small crack since the sectional shape of the larger crack has an inverse tapered shape, which is expected for the development of a small crack. These cavities are due to incomplete adhesion, air bubbles, and dust introduced in the lamination process. As shown in Figure 25b, as the temperature decreases in the cooling process, in-plane tension is created and stress concentrates around the cavity, leading to a crack. Hence, in the present shape design, the convex corners in the 2.5-D structure are rounded and their number is reduced to decrease incomplete adhesion. The remaining problem is the reduction in air bubbles and dust introduced in the lamination process; this problem will be considered in future work.

### 4.2. Size Reduction in Drivetrain

**Figure 26:**
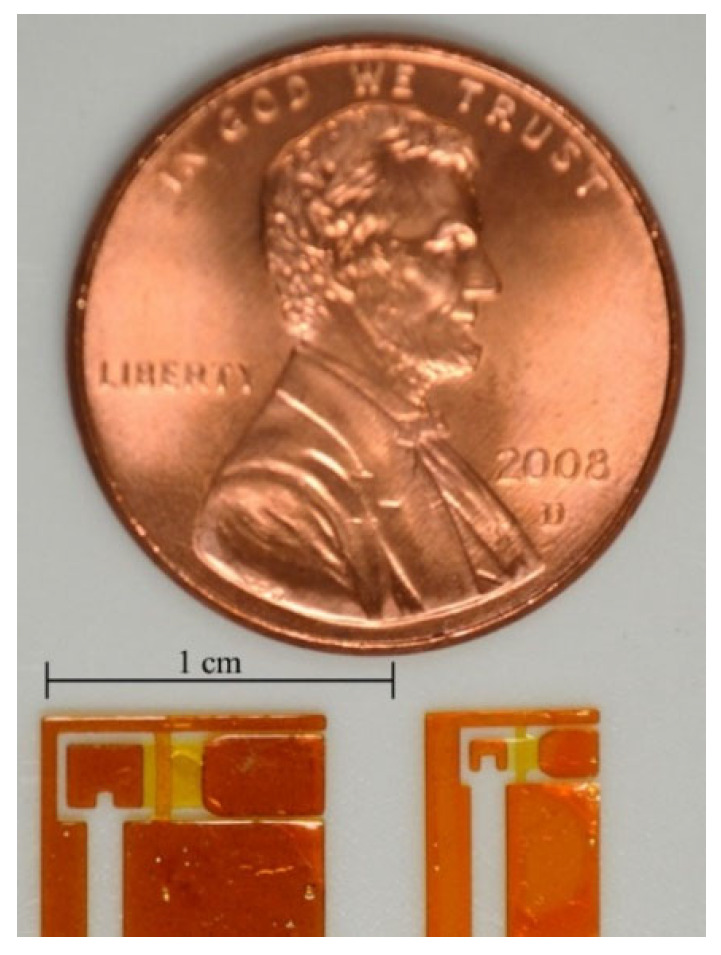
Fabrication results for large and small drivetrains.

This section demonstrates that the proposed 2.5-D structure approach can further miniaturize FWAVs. For this purpose, the small drivetrain obtained using the proposed design method as a miniaturization of the large drivetrain is fabricated using the proposed fabrication method of polymer MEMS micromachining. Figure 26 shows the fabrication results for one-wing drivetrains (dimensions shown in Table 1). As shown, the size of the drivetrain was successfully reduced.

**Figure 27:**
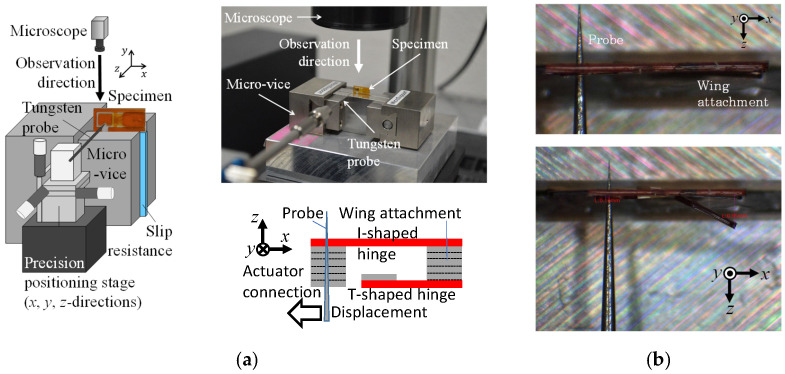
(**a**) Experimental setup of static test for drivetrain. (**b**) Observations of static test in initial state (upper figure) and after deformation due to forced displacement statically imposed by probe (lower figure).

Figure 27a shows the experimental apparatus used for the static test and the test procedure. A forced displacement is quasi-statically applied to the actuator connection using the probe. Figure 27b shows the observations of the static test. A large flapping angular displacement of the wing attachment is produced by the small forced displacement imposed on the actuator connection.

**Figure 28:**
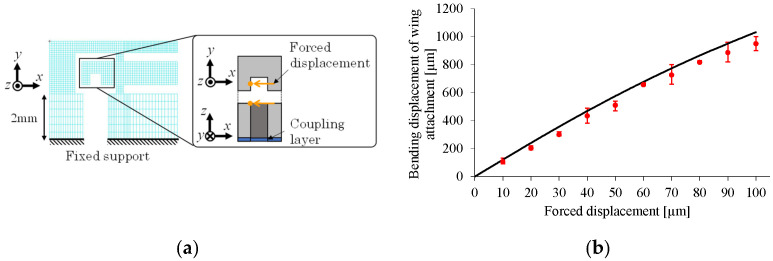
FE analysis results for small drivetrain. (**a**) FE modeling and boundary conditions. (**b**) Bending displacement of wing attachment produced by force displacement (black line: numerical result; red circles: experimental results).

Figure 28a shows the FE model corresponding to the present experiment. In the numerical setup, the coupling layer is considered as the base layer, whose thickness is set to 7.6 μm. Young’s modulus is set to 2.5 GPa for each PI layer and 3.5 GPa for the coupling layer. Figure 28b shows a comparison between the experimental and numerical results. In the experiment, each forced displacement (ranging from 10 to 100 μm in 10 μm increments) is applied to the specimen three times. The numerical results agree well with the experimental results, confirming the successful fabrication of the small drivetrain. These results indicate that the proposed 2.5-D structure approach further miniaturizes the drivetrain, leading to further miniaturization of FWAVs.

### 4.3. Fabrication of Complex Drivetrain

**Figure 29:**
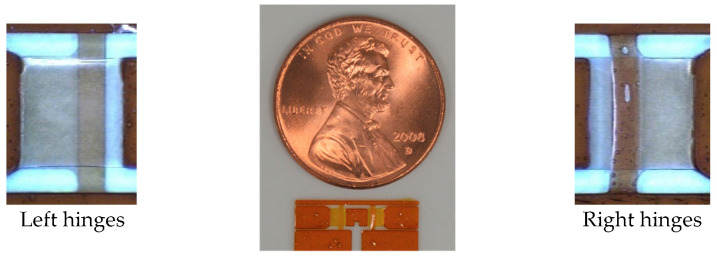
Fabrication results for two-wing drivetrain.

This section demonstrates the fabricability of complex FWAVs using the proposed 2.5-D structure approach. For this purpose, a two-wing drivetrain is fabricated using polymer MEMS micromachining. The difficulty of fabricating this 2.5-D structure is the requirement for simultaneous construction of two sets of parallel T- and I-shaped hinges, which are rotationally symmetrical with each other with respect to the center of their section. In the upper layer, one set has a T-shaped hinge and the other set has an I-shaped hinge. The T- and I-shaped hinges have different fabrication difficulties. Figure 29 shows the fabrication results (dimensions are based on the large drivetrain in Table 1). As shown, the complex drivetrain was successfully fabricated.

**Figure 30:**
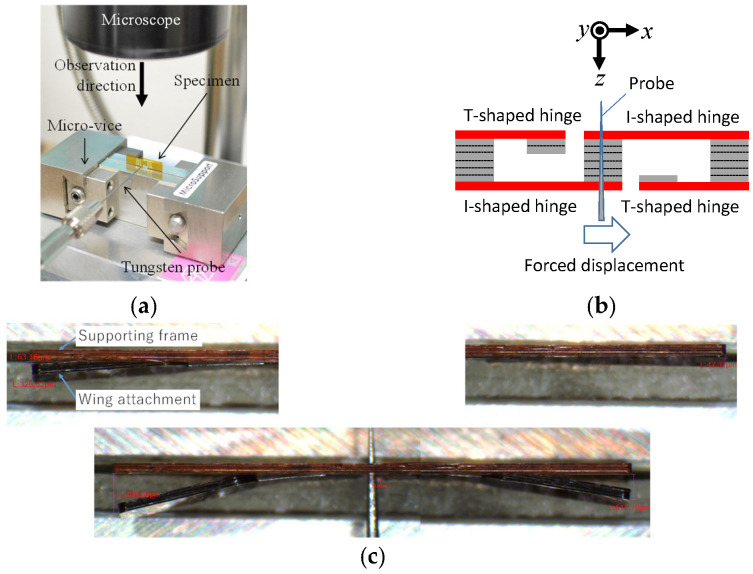
(**a**) Experimental setup. (**b**) Forced displacement quasi-statically applied to the actuator connection using the probe. (**c**) Detailed experimental process, where the upper left and right figures show the initial states of the left and right wing attachments, respectively, and the lower figure shows the state after deformation due to (**b**).

Figure 30a shows the specimen placed in the static test apparatus shown in Figure 27a. The forced displacement is quasi-statically applied to the actuator connection using the probe, as shown in Figure 30b. Then, the structure is deformed, as shown in Figure 30c. As shown, the elastic hinges are deformed such that they flap both the left and right wing attachments.

**Figure 31:**
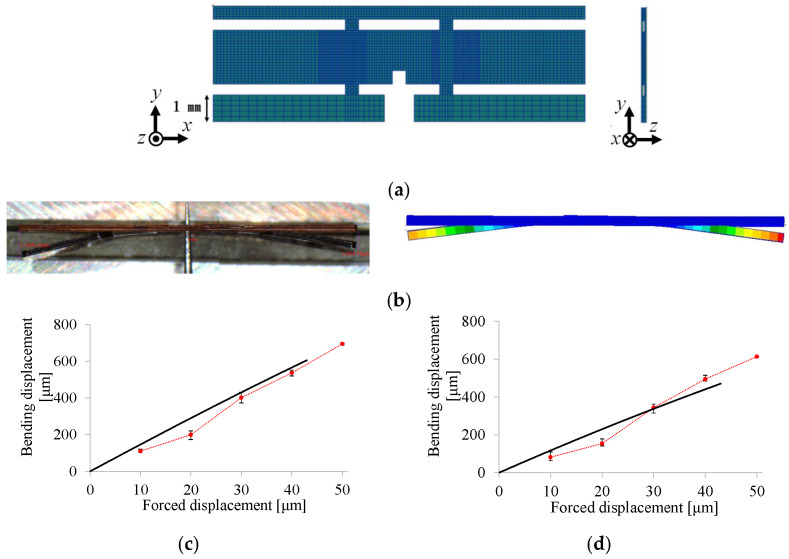
FE analysis results for two-wing drivetrain. (**a**) FE model corresponding to experiment. (**b**) Results of experimental and numerical static test (top: experimental results; bottom: numerical results). Numerical and experimental displacements for (**c**) left and (**d**) right wing attachments (black lines: numerical results; red circles: experimental results).

Figure 31a shows the FE model corresponding to the experiment. In the numerical setup, the coupling layer is considered as the base layer, whose thickness is set to 7.6 μm. Young’s modulus is set to 2.5 GPa for each PI layer and 3.5 GPa for the coupling layer. Figure 31b shows the experimental and numerical results of the static test. Their overall trends regarding deformation are similar to each other. Figure 31c,d show the relationships between the forced displacement imposed on the actuator connection and the resulting bending displacement at the tip of the wing attachment for the left and right sides, respectively. The numerical results agree well with the experimental results. These results indicate that the fabricability of the drivetrain with a complex 2.5-D structure is feasible. Complex FWAVs can thus be fabricated using the proposed 2.5-D structure approach.

### 4.4. Dynamic Test for Wing Flapping with Sufficient Stroke Angle

This section demonstrates that an insect-mimetic FWAV obtained using the proposed 2.5-D structure approach has a sufficient flapping ability. For this purpose, a one-wing large drivetrain is resonantly driven using the piezoelectric bimorph. A flapping motion similar to that of actual small insects is demonstrated.

#### 4.4.1. Experimental Setup

**Figure 32:**
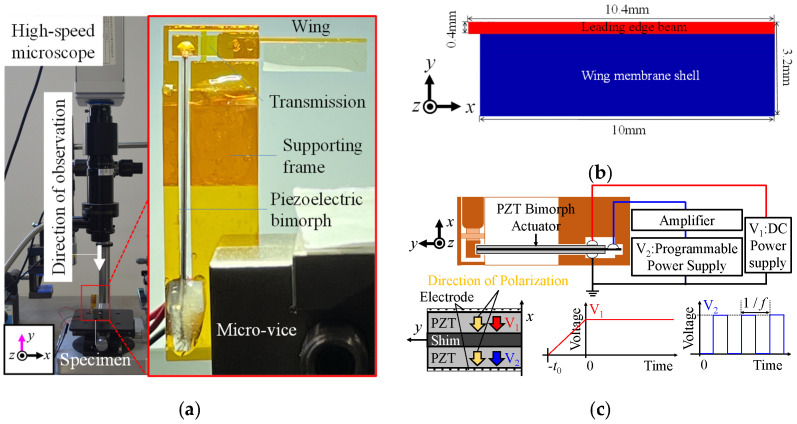
(**a**) Experimental apparatus for dynamic test and specimen. (**b**) Model wing. (**c**) Schematic diagram of circuit and voltages applied to bimorph.

Figure 32a shows the experimental apparatus used for the dynamic test. As shown on the right-hand side of this figure, the piezoelectric bimorph and the model wing are attached to the one-wing large drivetrain and the lower right edge is fixed to the ground. As shown on the left-hand side of this figure, the wing’s flapping motion is recorded using a high-speed microscope (8000 frames per second and VGA output) in the longitudinal direction of the drivetrain.

As shown in Figure 32b, the model wing consists of a rectangular leading-edge beam and the wing membrane, which are fabricated simultaneously using the same polymer MEMS micromachining as that used for the drivetrain. Figure 32c shows the electric configuration for driving the piezoelectric bimorph sinusoidally. A constant voltage *V*_1_ = V* is applied between the upper and lower electrodes and a periodic voltage with a rectangular waveform and amplitude *V*_2_ = V* is applied between the upper electrode and the middle shim. V* is periodically and alternatively applied to the upper or lower piezoelectric layer such that the electric field is always directed in the polarization direction, making the piezoelectric bimorph vibrate sinusoidally.

#### 4.4.2. Results and Discussion

**Figure 33:**
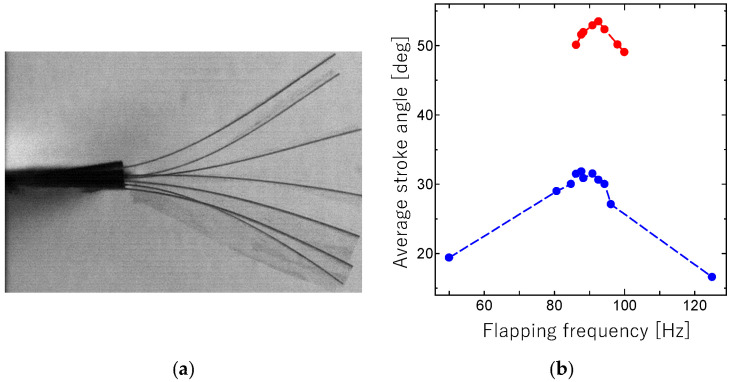
(**a**) Sequential movement of wing flapping during half-stroke (flapping frequency: 92.6 Hz; applied voltage: 100 V). (**b**) Average stroke angle versus flapping frequency (blue: applied voltage of 50 V; red: applied voltage of 100 V).

Figure 33a shows the sequential movement of the flapping wing during a half-stroke for applied voltage V* = 100 V and resonant frequency f* = 93 Hz. As shown, a sufficient stroke angle is applied to the wing using the proposed resonant drive method. Figure 33b shows the average stroke angle versus flapping frequency.

The maximum stroke angle Φ* is 32° for V* = 50 V and f* = 88 Hz, and 53° for V* = 100 V and f* = 93 Hz. These values are comparable to those for insects with indirect flight muscles. The present results correspond to the design solution of the first vibration mode for the proposed resonant drive of the flapping system since f* is in the predicted range of the natural frequency. These results indicate that the proposed flapping system has a sufficient flapping ability.

## 5. Concluding Remarks

In this study, we proposed an approach for miniaturizing FWPAVs that consists of the design and fabrication of a 2.5-D structure. The proposed approach was applied to the flapping system for FWAVs. This study fabricated a prototype flapping system for insect-mimetic FWAVs using MEMS micromachining without any post-assembly of structural components. It was demonstrated that the flapping system could be further miniaturized while maintaining its flapping ability. A complex 2.5-D structure was fabricated using polymer MEMS micromachining. Different from previous studies, no post-assembly process was used to develop the drivetrain in this study. The flapping system produced wing flapping motions that had a magnitude and a frequency comparable to those of actual small insects. Different from previous studies, this motion was achieved without added mass in this study. These results indicate that the proposed approach can further miniaturize FWPAVs. In future work, we will evaluate lift and demonstrate actual flight using the proposed MEMS flapping system. Furthermore, the yield or reproducibility of the proposed fabrication method will be comprehensively studied for the further development of insect-mimetic FWPAVs.

## Figures and Tables

**Table 1:** Dimensions of large and small flapping systems.

Parameter	Large	Small
*L* [mm]	20	10
*L*_A_ [mm]	8.9	5.55
*D*_A_ [mm]	30.2	19.2
*W* [mm]	3	2
*t*_p_ [mm]	0.2	0.2
*t*_m_ [mm]	0.1	0.1
*H* [mm]	6.5	6.5
*t*_H_ [mm]	2.5	2.5
*L*_s_ [μm]	500	300
*L*_HT_ [μm]	1000	400
*L*_HI_ [μm]	1800	1000
*h*_g_ [μm]	200 (240) ^1^	104 (146) ^2^
*D*_T1_ [μm]	2000	1000
*D*_T2_ [μm]	400	400
*R* [μm]	200	200

^1^ The 200 μm (240 μm) gap is made of 5 (6) layers of PI sheets with 40 μm thickness in the one-wing system (two-wing system). ^2^ The 104 μm (146 m) gap is made of 4 layers of PI sheets with 26 μm thickness (1 layer of PI sheets with 26 μm thickness and 3 layers of PI sheets with 40 μm thickness).

**Table 2:** Material properties.

Property	PI	PZT	Shim	Epoxy Resin
Young’s modulus [GPa]	2.5	61.0	140	3.0–6.0
Poisson’s ratio	0.289	0.31	0.36	0.30
Mass density [kg/m^3^]	1420	7820	8730	1100–1400

## Data Availability

The datasets analyzed during the current study are available from the corresponding author upon reasonable request.
